# Real samples sensitive dopamine sensor based on poly 1,3-benzothiazol-2-yl((4-carboxlicphenyl)hydrazono)acetonitrile on a glassy carbon electrode

**DOI:** 10.1038/s41598-024-65192-0

**Published:** 2024-07-18

**Authors:** Hesham M. Alsoghier, Mohamed Abd-Elsabour, Abdulrahman G. Alhamzani, Mortaga M. Abou-Krisha, Hytham F. Assaf

**Affiliations:** 1 Chemistry Department, Faculty of Science, South Valley University, Qena, 83523 Egypt; 2 Chemistry Department, Faculty of Science, Luxor University, Luxor, 85951 Egypt; 3 Chemistry Department, College of Science, Imam Mohammad Ibn Saud Islamic University (IMSIU), 11623 Riyadh, Saudi Arabia

**Keywords:** Dopamine, Benzothiazole azo dye, Modified glassy carbon electrode, Electropolymerization, Cyclic voltammetry, Chemistry, Analytical chemistry, Electrochemistry, Organic chemistry, Polymer chemistry

## Abstract

Herein, a novel electrochemical sensor that was used for the first time for sensitive and selective detection of dopamine (DA) was fabricated. The new sensor is based on the decoration of the glassy carbon electrode surface (GC) with a polymer film of 1,3-Benzothiazol-2-yl((4-carboxlicphenyl)hydrazono)) acetonitrile (poly(BTCA). The prepared (poly(BTCA) was examined by using different techniques such as ^1^H NMR, ^13^C NMR, FTIR, and UV-visible spectroscopy. The electrochemical investigations of DA were assessed using cyclic voltammetry (CV) and differential pulse voltammetry (DPV). The results obtained showed that the modifier increased the electrocatalytic efficiency with a noticeable increase in the oxidation peak current of DA in 0.1 M phosphate buffer solution (PBS) at an optimum pH of 7.0 and scan rate of 200 mV/s when compared to unmodified GC. The new sensor displays a good performance for detecting DA with a limit of detection (LOD 3σ), and limit of quantification (LOQ 10σ) are 0.28 nM and 94 nM respectively. The peak current of DA is linearly proportional to the concentration in the range from 0.1 to 10.0 µM. Additionally, the fabricated electrode showed sufficient reproducibility, stability, and selectivity for DA detection in the presence of different interferents. The proposed poly(BTCA)/GCE sensor was effectively applied to detect DA in the biological samples.

## Introduction

Dopamine (DA) belongs to the catecholamine family, which plays a pivotal role as a neurotransmitter in the central nervous, hormonal, renal, and cardiovascular systems^1–4^. DA, known as 3,4-dihydroxy phenylethylamine, is associated with neuronal functions such as cognition, motivation, attention, learning, memory, and so on^3,5–7^. DA acts as intravenous medication, which increases heart rate and blood pressure^3,8–11^. DA Lacking in the body may lead to Parkinson's and Alzheimer's diseases^12,13^. These not only, but also schizophrenia, Tourette's syndrome, and hyperactive disorder are related to insufficient concentrations of DA^14,15^. Therefore, sensitive determination of DA is vitally necessary In vitro and In vivo.

In recent years, various analytical methods have been developed to quantitative and qualitatively analyze DA either in human biological samples and its pharmaceutical formulation, including high-performance liquid chromatography^16^, capillary electrophoresis^17,18^, UV-visible spectrometry^19^, fluorimetry^20^, enzymatic^21^, and electrochemical methods^22–26^. The electrochemical methods are interesting alternative techniques for pharmaceutical drugs because of their unique properties such as simplicity, low cost, rapid response, and high sensitivity^27–30^. Glassy carbon electrodes (GCE) are considered an important bar electrode that has been widely studied thanks to the relatively low cost, chemical inertness, and wide potential window^31^. However, it was noticed that the redox of DA at bare electrodes requires high overpotentials. In addition, the oxidation potentials of some biological molecules, such as uric acid and ascorbic acid, are close to that of DA on unmodified electrodes, resulting in poor selectivity of DA^32^. Thus, it is essential to develop the efficiency of the bar GCE to be distinguished by reliable, selective, and sensitive, as well as characterised by the high thermal stability, excellent redox conductivity with high electrocatalytic effect, and large active-surface area of DA detection. To achieve this target, a thin polymeric film is an ideal material for electrode surface modification^3,8,32^.

Recently, benzothiazole-based azo dyes are found to be quite interesting because of their wide demands in dye-sensitized solar cells^33–35^, dyeing agents for foodstuff^36^, textiles^36^, liquid crystal displays^37,38^ and electro-optical devices^37–39^, electrophosphorescent emitters^40^, NLO^34,41^, redox properties^42^, chemosensors^43^, optics^34,41,44^, structure-activity exchanges^45^, pharmacology and biology^46,47^. The polymer films of an organic material exhibit high conductivity during redox reactions due to significant π-electron delocalization in their backbone. Furthermore, azo dyes have a high surface area for biomolecules to be immobilized, thereby increasing the number of binding sites available for the detection of a specific analyte and the ability to facilitate electron transmission^48,49^.

In this paper, we adopt a new strategy for fabricating a new electrochemical sensor based on electrochemical polymerization of 1,3-Benzothiazol-2-yl((4-carboxlicphenyl) hydrazone) acetonitrile (BTCA) on the surface of a glassy carbon electrode (GCE) for a first time to determine DA in biological samples. The polymerization mechanism of BTCA was investigated via the suggested proton transfer mechanism (Scheme 2). The electrochemical behavior of DA on the surface of the poly(BTCA)/GCE sensor was explored through different voltammetric techniques, such as cyclic voltammetry (CV) and differential pulse voltammetry (DPV). The data obtained refers to the success of using GCE modified by the polymer film of BTCA toward DA oxidation, where the results demonstrate a giant enhancement in the peak current, high selectivity, and sensitivity. Hence, the poly(BTCA)/GCE sensor shows good electrocatalytic activity with DA in real samples.

## Experimental

### Chemical reagents and solutions

Dopamine hydrochloride (DA, 98.0%), hydrochloric acid (HCl, 32.0%), and sulfuric acid (H_2_SO_4_, 98.0%) were obtained from Sigma-Aldrich. Sodium hydroxide, sodium carbonate, and bicarbonate were gained from El-Nasr Pharmaceutical Chemicals (Egypt) with a purity of 99.0%. In our research, all chemicals were of analytical grade unless otherwise stated and were used straight without any further refinement. All solutions were freshly set using ultrapure water (18.2 MΩ/cm) at chamber temperature. A buffer solution of 0.1 M Na_2_HPO_4_–NaH_2_PO_4_ (PBS, pH 7.0) was obtained from El Nasr Pharm. Chem. Co (ADWIC), 98% purity, was used as the supporting electrolyte. 1.0 M of HCL and NaOH (El Nasr Pharm. Chem. Co (ADWIC), 96% purity) solutions were used to regulate the chosen pH.

### Apparatus and cell

The electrochemical trials were achieved by using an EG&G Princeton applied research potentiostat/galvanostat model 263A (USA) in a three electrodes micro-cell (model K0264) in which a pure platinum rope (model K0266) and Ag/AgCl saturated with KCl 3.0 M (model K0265) were operated as auxiliary and reference electrodes respectively. While a bare GCE (0.3 mm diameter model G0229) and the poly(BTCA)/GCE were used as the working electrodes. A CyberScan pH 500 Meter (Euteoh-India) was engaged to regulate pH values. To ensure the structure of (BTCA)Azo dye, many apparatuses were used, such as UV-visible absorption spectra were monitored on a Shimadzu 2401PC spectrophotometer (equipped with a Julabo F30 ultra thermostat with an accuracy of − 0.51 C) within the wavelength range of 200–800 nm using a thermostat (T = 25.0 + 0.2 C) 1 cm matched quartz cells. The ^1^H and ^13^C NMR spectra were recorded on a Bruker Avance II. All the measurements were achieved at room temperature (≈ 251 C) in the NMR Unit, Faculty of Pharmacy, Mansoura University.

### Synthesis of 1,3-benzothiazol-2-yl((4-carboxlicphenyl)hydrazono) acetonitrile (BTCA) azo dye

**Scheme 1:**
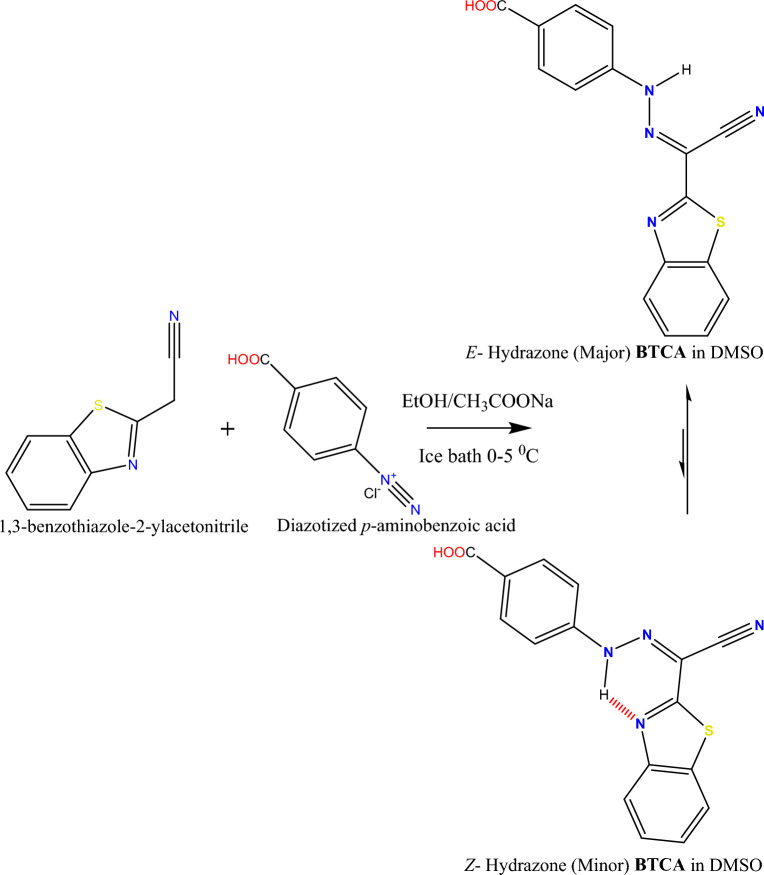
Synthesis of **BTCA** azo dye.

BTCA azo dye (Scheme 1) was prepared according to the procedure described in the literature^33,34,39,41,50^. Concisely, to an ethanolic solution of 1,3-benzothiazole-2-ylacetonitrile (0.01 mol) in the existence of sodium acetate (≈ 0.01 mol) in an ice bath, a solution of diazotized *p*-aminobenzoic acid was added drop wisely with vigorous stirring at 0–5 °C for one hour. The target BTCA precipitate was filtered off and washed with water and cold ethanol. The solid BTCA azo dye was recrystallized from ethanol and dried in a vacuum over silica gel.

### Fabrication of the poly(BTCA)/GCE

Before the modification of the GCE surface, a 3 µm of alumina powder was used successively to polish a GCE to a mirror-like smoothness surface, then rinsed with distilled water and ultrasonically cleaned with water/ethanol for 10 min. In addition, the GCE was electrochemically cleaned by cycling the electrode potential from − 1.0 to 1.0 V (vs. Ag/AgCl) in 1.0 M H_2_SO_4_^29,51^ till a stable voltammogram was obtained. CV was employed to carry out the polymerization of 1.0 mM BTCA monomer in 0.1 M of PBS: MeOH (v:v, 25:25) for 13 cycles at a negative potential between 0.0 and − 1.8 V with a scan rate of 100 mV/s. Finally, the fabricated sensor was meticulously rinsed with double distilled water and stored at room temperature.

### Real sample preparation

Human blood serum was collected from the South Valley University hospital and then diluted with PBS (pH = 7.0). Urine samples of the healthy specimen were analyzed immediately after collection. Three milliliters of fresh samples was added to 4 ml methanol to precipitate any protein that might be present in the sample.. Then, the solution was centrifuged for 10 min at 2500 rpm. The supernatant was directly diluted to 25 ml with PBS (pH = 7.0) in the electrochemical cell (https://www.sciencedirect.com/topics/materials-science/electrochemical-cell). The real samples were spiked with different amounts of DA. The concentration of DA was determined by the standard addition method, and the average recovery percentage was calculated^30,52^.

## Results and discussion

### Characterization of the synthesized 1,3-benzothiazol-2-yl((4-carboxlicphenyl) hydrazono) acetonitrile (BTCA)

**Figure 1:**
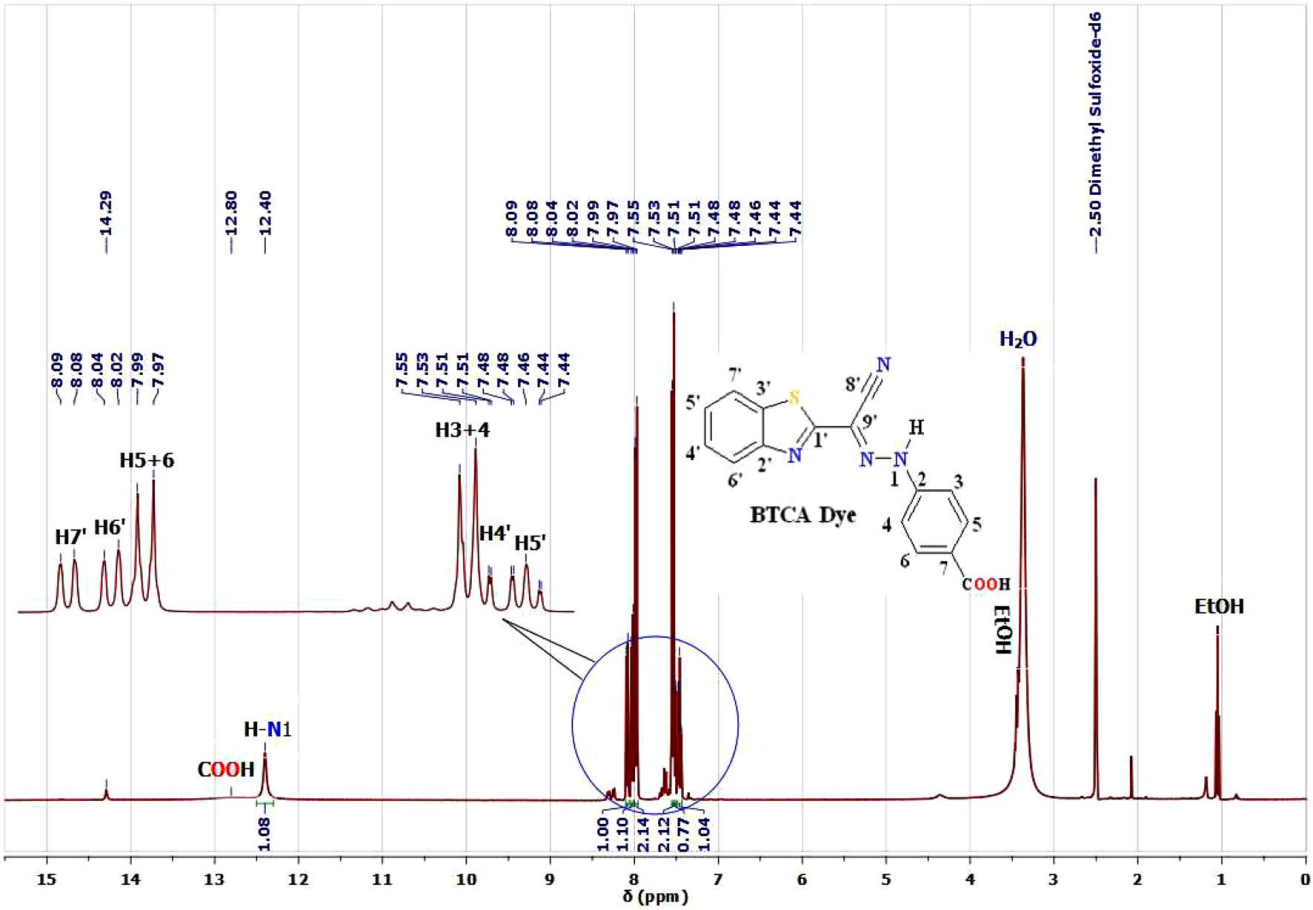
^1^H NMR spectrum of BTCAazo dye in DMSO-d6.

**Figure 2:**
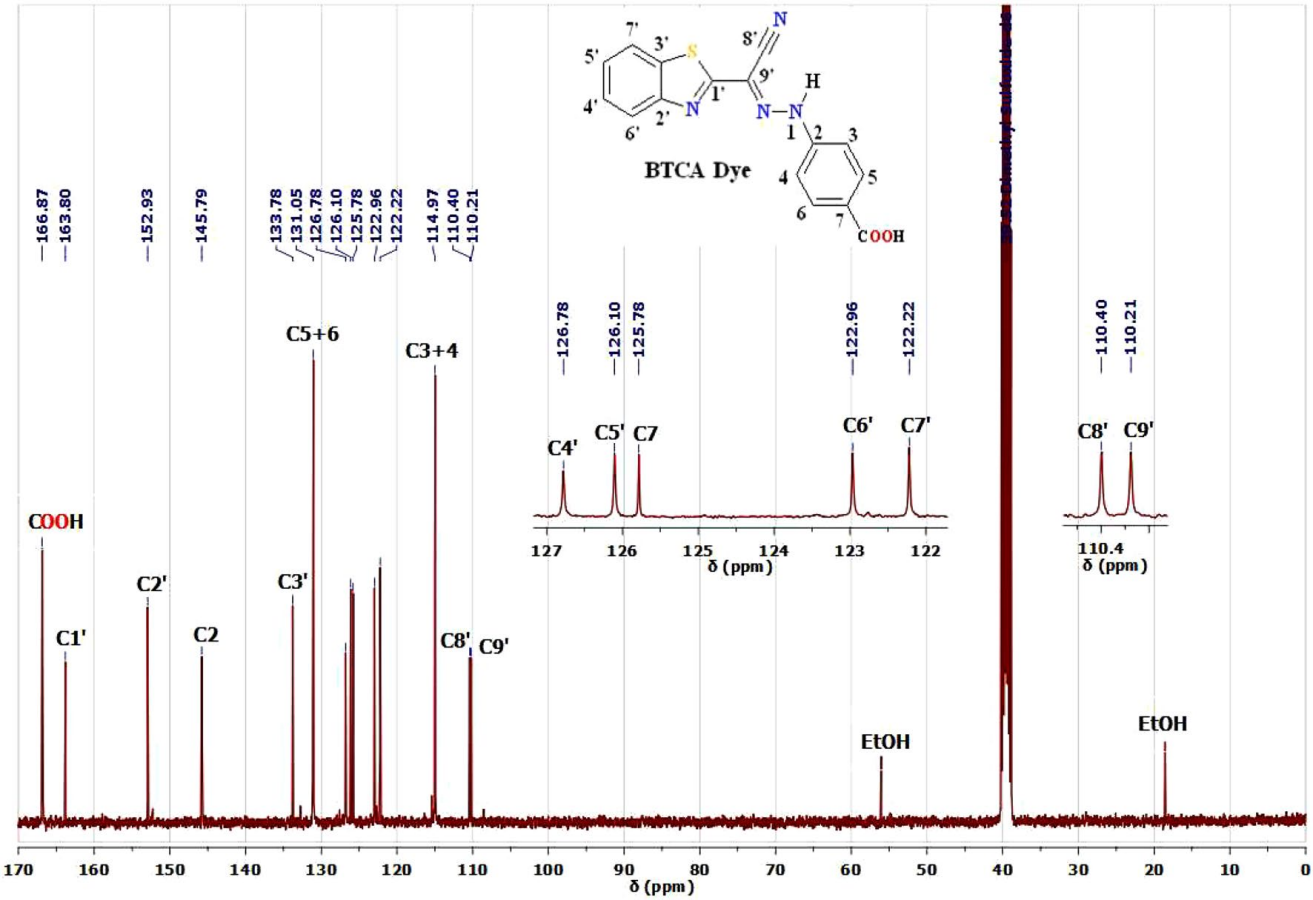
^13^C NMR spectrum of BTCA azo dye in DMSO-d6.

^1^H and ^13^C NMR are characterizing tools for proving the chemical structure of our BTCA azo dye. The ^1^H and ^13^C NMR spectra of BTCA dye in DMSO-d6 are represented in Figs. 1 and 2. The proton NMR spectrum of BTCA dye (Fig. 1) showed broad bands at chemical shift (δ 12.40 and 14.29 ppm) that could be assigned to NH protons^33,34,39,41,50^ of the *E*-hydrazone (major) in equilibrium with *Z*-hydrazone (minor) forms (Scheme 1), respectively. In similarity, the accompanied COOH proton exists as a broad peak at chemical shift (δ 12.80 ppm).

In consistency with the literature, the aromatic protons of the BTCA dye can be divided into two patterns: four benzothiazole protons pattern (δ(^1^H) = 7.46 and 8.06 ppm) and four phenyl protons pattern (δ(^1^H) = 7.54 and 7.98 ppm). Four benzothiazole ring protons pattern consists of two downfield doublets peaks appointed to protons H7' (δ(^1^H) = 8.08–8.09 ppm) and H6' (δ(^1^H) = 8.02–8.04 ppm) as well as two up field triplets signals can be assigned to H4' (δ(^1^H) = 7.51–7.53 ppm) and H5' (δ(^1^H) = 7.44–7.48 ppm) as shown in Fig. 1. However, the phenyl moiety protons pattern shows two doublets peaks (A_2_B_2_ system). The first doublet peak is assigned to the two protons H3 + 4 (δ(^1^H) = 7.53–7.55 ppm), and the second doublet peak to the two protons H5 + 6 (δ(^1^H) = 7.97–7.99 ppm) as presented in Fig. 1.

Examination and characterization of the ^13^C NMR spectrum of the dye BTCA in (CD_3_)_2_S = O were presented in Fig. 2 and Table 1. Fourteen signals of carbons can be identified, and all carbons resonate at region (δ(^13^C) = 110.21–166.87 ppm). In consistence with the upper ^1^H NMR spectra of BTCA dye, we did not observe any aliphatic carbon peak in the ^13^C NMR spectra. This gives us a shred of sharp evidence to neglect the probability of the existence of an azo tautomeric form of this dye and confirm the existence of *E*-hydrazone form (major)^34,39,41,50^.

**Table 1:** ^1^H and ^13^C NMR assignments of the BTCA dye in (CD_3_)_2_S = O.

H/C No.	**BTCA** dye	
	δ(^1^H)	δ(^13^C)
1'	–	163.80
2'	–	152.93
3'	–	133.78
4'	7.53–7.51 (m, 1H)	126.78
5'	7.48–7.44 (m, 1H)	126.10
6'	8.03 (d, J = 7.6 Hz, 1H)	122.96
7'	8.09 (d, J = 7.3 Hz, 1H)	122.22
8'	–	110.40
9'	–	110.21
2	–	145.79
3,4	7.54 (d, J = 8.8 Hz, 2H)	114.97
5,6	7.98 (d, J = 8.8 Hz, 2H)	131.05
7	–	125.78
1N-H hydrazone	12.40 (s, 1H)	–
*p*-COOH	12.80 (b, 1H)	166.87

**Figure 3:**
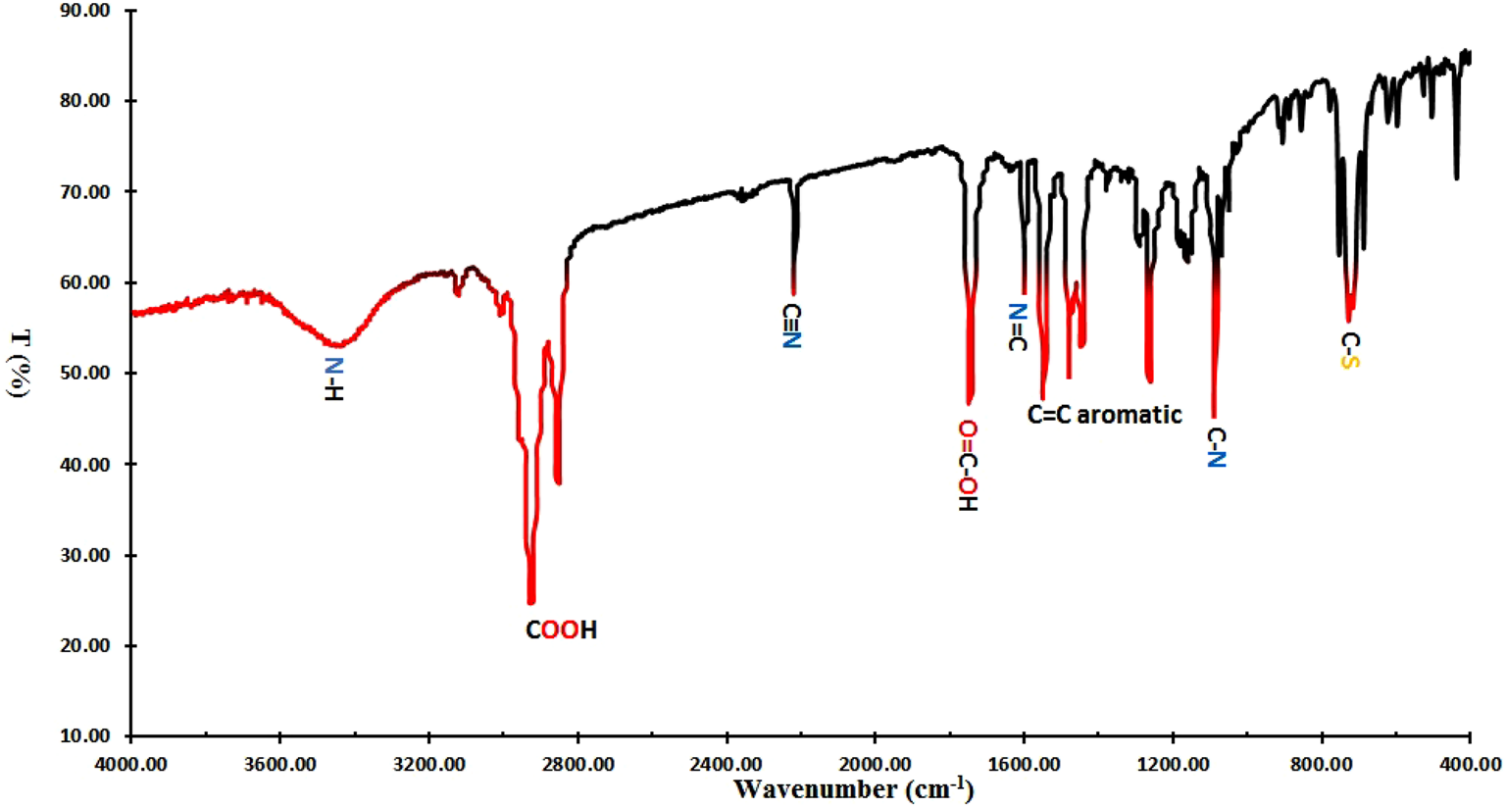
FT-IR spectrum of BTCA azo dye in KBr disk.

Our FT-IR spectrum (Fig. 3) of **BTCA** dye shows unique bands at 3420 cm^−1^ (N–H), 2920–2850 cm^−1^ (COO-H), 2220 cm^−1^ (C≡N), 1740 cm^−1^ (O=COH), 1600 cm^−1^ (N=C), 1550 cm^−1^ (C=N–N–), 1480–1260 cm^−1^ (C=C Aromatic), 1090 cm^−1^ (C-N), and 714 (C-S). These bands confirm the suggested *E*-hydrazone chemical structure of BTCA dye.

Finally, our IR, UV-Visible (Fig. S5), and NMR data confirmed the existence of the *E*-hydrazone form (major).

### Electropolymerization of 1,3-benzothiazol-2-yl ((4-carboxlicphenyl) hydrazono) acetonitrile (BTCA) on the GCE surface

**Figure 4:**
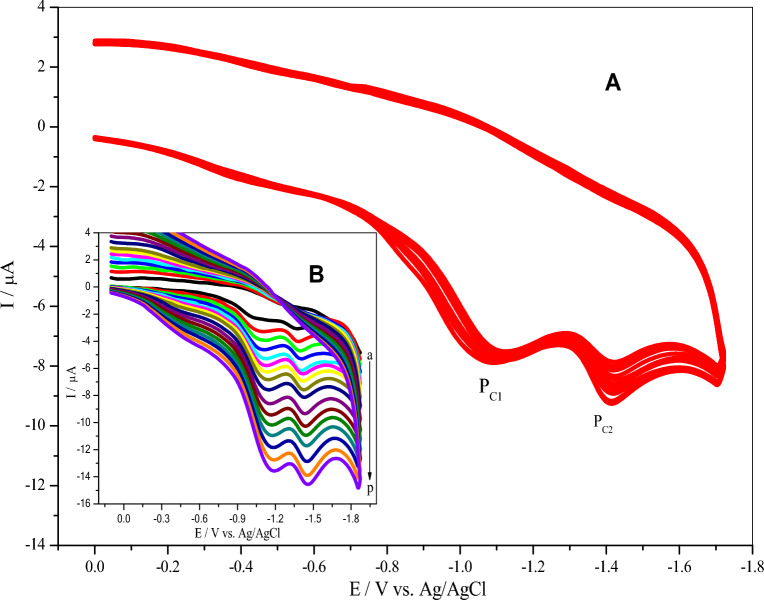
(**A**) Continuous CVs for the electropolymerization of 1.0 mM BTCA in 0.1 M PBS: MeOH on the GCE surface at a scan rate of 100 mV/s for 13 cycles. (**B**) Inset shows CVs of the poly (BTCA)/GCE in 0.1 M PBS (pH 7.0) at different scan rates (20–450 mV/s).

**Scheme 2:**
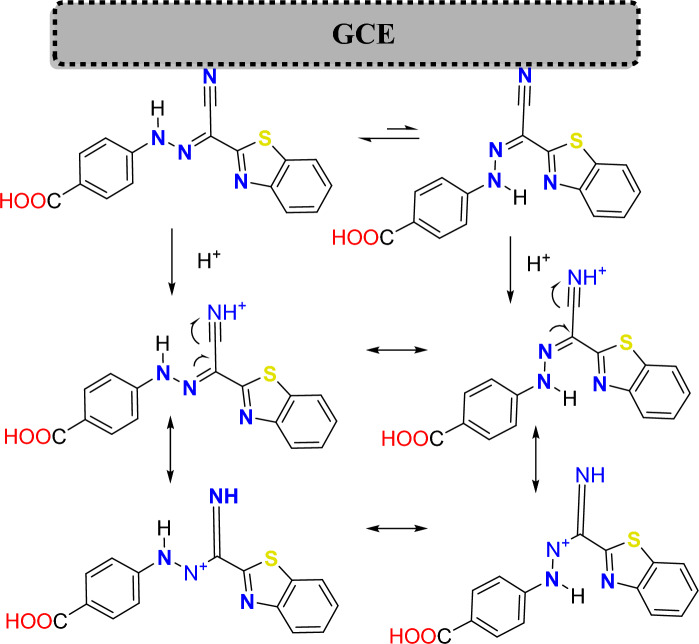
Suggested proton transfer mechanism of **BTCA** on GCE surface.

Herein, thirteen successive CVs were performed in the potential range between 0.0 and − 1.8 V for 1.0 mM of **BTCA** in 0.1 M PBS (pH 7.0) on the GCE surface at a scan rate of 100 mV/s (Fig. 4A). As observed during the first potential scan, two reduction peaks appear at (P_C1_) − 1.11, and (P_C2_) − 1.42 V, respectively. These two reduction peaks may be attributed to bearing a benzothiazole acceptor group linked to the azo bridge^53–55^. Both peak currents were getting larger and larger with increasing the number of cycles, indicating additional electroactive **BTCA** thin film deposition. Also, the gradual shift of their potential towards positive values accelerates the electron transfer from the solution to the electrodeposited **BTCA** thin film. This behavior reflects the continuous growth of the polymer film of **BTCA** onto the GCE surface, as shown in Scheme 2. During the thirteen cycles, the nature of the voltammogram was distorted because of a constraint in electron transfer caused by the increased thickness of the polymer film. Thus, thirteen cycles were chosen to give a suitable thickness for the polymeric thin film of **BTCA** on the GCE surface. Inset Fig. 4B displays the CVs of the poly (**BTCA**)/GCE in 0.1 M PBS (pH 7.0) at different scan rates in the range of 20–450 mV/s. The peak currents were found to increase with the increase in the scan rate, which indicates surface-confined reaction kinetics^48,56–58^.

The presumable electropolymerization mechanism is shown in Scheme 2. Due to the existence of *E*-hydrazone (major) in equilibrium with *Z*-hydrazone (minor) forms, both **BTCA** forms can be reduced on the surface of the glassy carbon electrode. **BTCA** dye has a high ability to gain proton from the solution (PBS pH 7.0) because of the presence of electron donor atoms (N & S)^13,48,59–61^. After proton gaining of both **BTCA** *E and Z* hydrazone forms in the solution, resonance can occur between both forms, as shown in Scheme 2. These various resonating structures are helpful in the adsorption and polymerization of **BTCA** dye on the glassy carbon electrode surface^59^.

### Electroactive surface area measurements

**Figure 5:**
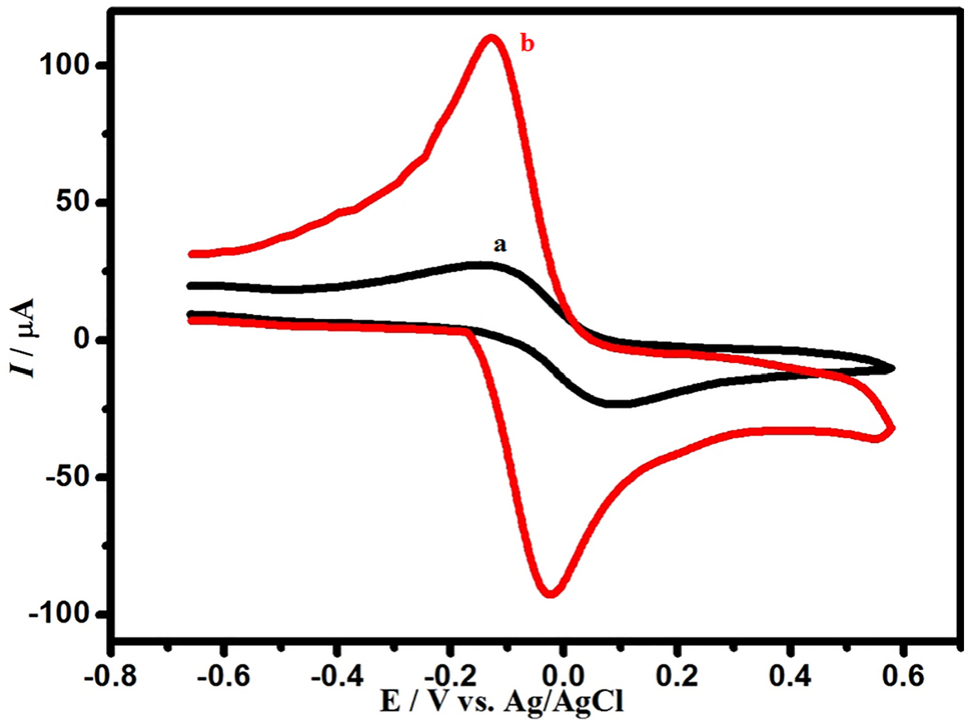
Cyclic voltammograms 1.0 mM of [Fe(CN)_6_]^3-/4-^ in KCl (0.1 M) solution at a scan rate of 50 mV at (a) bare GCE (black line), (b) poly(BTCA)/GCE (red line) 3.4. Electrochemical sensing of DA at poly(BTCA)/GCE.

This is to ensure the selected modifier is more effective. The electroactive surface area for both bar GCE and poly (BTCA)/GCE must be measured. That can occur by applying a cyclic voltammetry technique for the two electrodes, using KCl (0.1 M) as a supporting electrolyte containing 1.0 mM [Fe(CN)_6_]^3−/4−^ with a scan rate of 50 mV/s as illustrated in (Fig. 5). The figure showed that both electrodes displayed a reversible redox. Additionally, the peak current intensity in case poly (**BTCA**)/GCE increased by about 4 times compared to bar GCE, this demonstrates how **BTCA** enhanced the efficiency of the modified electrode and decreased the charge-transfer resistance, Thus refers to the presence of **BTCA** on the surface of glassy carbon increase the electrocatalytic activity of the modified electrode and can be applied successfully for the appropriate analytical applications. The active surface area (A) for both applied electrodes was calculated using the Randles–Sevcik formula^30,62,63^.

$$\left( {{\text{Ip}} = \left( {26.9 \times 10^{5} } \right){\text{n}}^{1.5} {\text{AD}}_{{\text{R}}}^{0.5}\upupsilon ^{0.5} {\text{C}}_{{\text{o}}} } \right)$$

where i_p_ refers to the anodic peak current, n is the electron transfer number, A is the surface area of the electrode, D_R_ the diffusion coefficient, C_0_ is the concentration of K_3_Fe(CN)_6,_ and v is the scan rate. For 1.0 × 10^−3^ mol L^−1^ K_3_Fe(CN)_6_ electrolyte, n = 1, D_R_ = 7.6 × 10^−6^ cm^2^ s^−1^. The active surface areas can be calculated. In bare GCE, the active surface is 0.0314 cm^2^ but in poly (**BTCA**)/GCE is 0.13 cm^2^, which means the surface of the modified electrode was greater four times than unmodified glassy carbon electrode.

**Figure 6:**
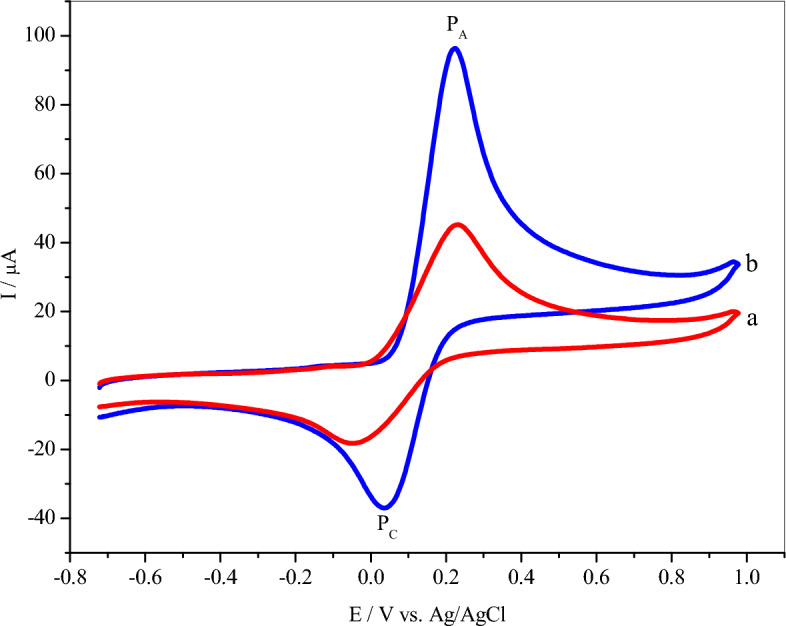
CVs of bare GCE (a) and the poly(BTCA)/GCE, (b) in 0.1 M PBS(pH 7.0) containing 35.0 μM DA at a scan rate of 50 mV/s.

**Scheme 3:**
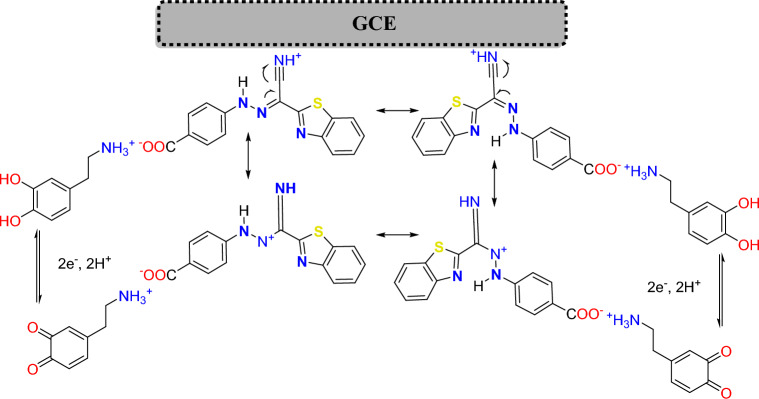
Suggested oxidation of Dopamine (DA) on poly(BTCA)/GCE surface.

The CVs of a bare GCE (curve a) and the poly (**BTCA**)/GCE (curve b) were reported in 0.1 M of PBS (pH = 7.0) containing 35.0 μM of **DA** with a constant scan rate (50 mV/s) as recorded in Fig. 6. At the bare GCE, **DA** exhibited poorly defined redox peaks in the given potential at + 0.233 V and − 0.047 V, respectively with a large ΔE_P_(E_PA_–E_PC_ = 280 mV). Meanwhile, a large increase in the redox peaks current of **DA** at the poly (**BTCA**)/GCE (curve b) vs. unmodified GCE (curve a). This not only but also the modified electrode achieved a lesser ΔE_P_ = 188, which is an indication that the oxidation process of **DA** is quasi-reversible^64^. Thus, facts are due to the presence of **BTCA** polymer on the GCE helping the electrochemical interaction between the carboxylic (COOH) active site of **BTCA** thin film and amine (NH_2_) moiety of **DA** (Scheme 3)^59,65^. This interaction occurs by receiving a proton from the carboxylic (COOH) group of **BTCA** to form ammonium (^+^NH_3_) cation of the **DA** NH_2_ group, as shown in Scheme 3. After this donor–acceptor interaction (COO^- +^NH_3_) on the surface of poly(**BTCA**)/GCE, a reversible redox process^51,66^ can be performed on the hydroxyl (OH) groups of **DA** catechol moiety (Scheme 3).

### Influence of solution pH

**Figure 7:**
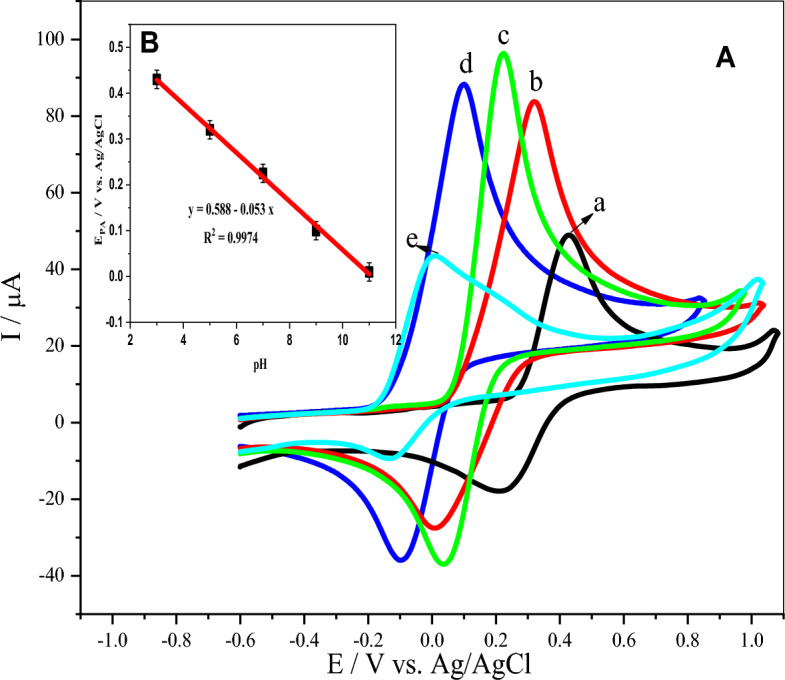
(**A**) CVs of 35.0 μM DA in 0.1 M PBS at the poly(BTCA)/GCE in various pHs (a toe are 3.0, 5.0,7.0,9.0 and 11.0) at a scan rate 50 mV/s. (**B**) Inset: plots of the effects of pH on the anodic peak potentials.

The pH of the solution produces a prominent impact on the electrical signal response of DA due to protons taking part in the electrooxidation reaction^67^. Hence, the influence of various pHs (3.0–11.0) was investigated using the poly (**BTCA**)/GCE for 35.0 μM DA in 0.1 M PBS at a scan rate of 50 mV/s (Fig. 7A). As can be seen, the anodic peak current of DA was intensified with an increase in pH from 3.0 to7.0 and then dropped till pH at 11.0. The highest current sensitivity is observed at pH 7.0 due to complete deprotonation and could be suitable for biological application. Therefore, a pH of 7.0 is an optimal value for subsequent experiments. Additionally, the effects of pH on the anodic peak potentials were recorded in the same range as shown in Fig. 7B. It's clear that the pH augmentation has resulted in a formal potential shift towards more negative values. Thus, the linear regression equation can be represented as the following.

$$E_{{{\text{pa}}}} \left( {\text{V}} \right) = 0.588 - 0.053\;{\text{pH}}\quad R^{2} = 0.9974.$$

The slope was found to be 53 mV/pH, which is too close to the Nernst value (59 mV/pH, 25 °C), which implies the electrooxidation of DA at the poly(**BTCA**)/GCE involved an equal number of electrons and protons^68^.

### Influence of scan rate

**Figure 8:**
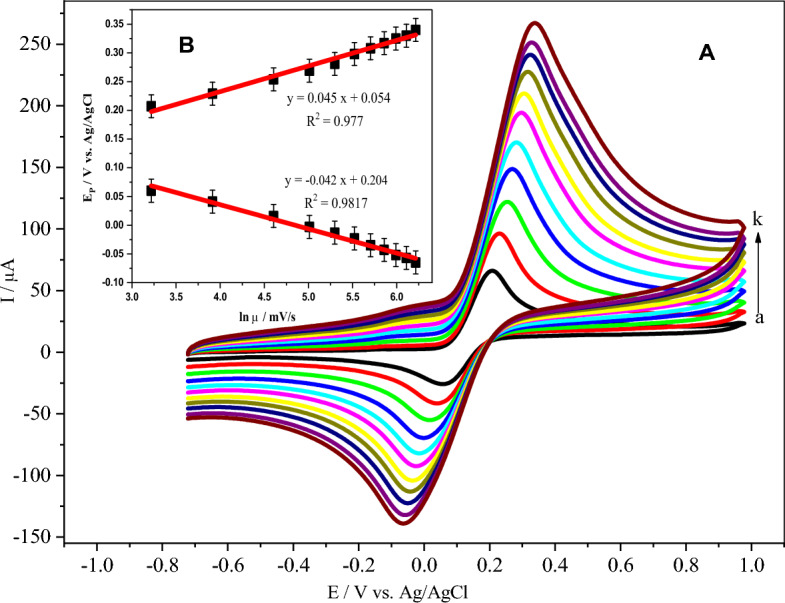
(**A**) CVs of 35.0 μM DA in 0.1 M PBS (pH 7.0) on the poly (BTCA)/GCE surface at various scan rates: (*a* to *k* are 25, 50, 100, 150, 200, 250, 300, 350, 400, 450, and 500 mV/s). (**B**) Inset: plots of the effects of the square root of scan rate on the anodic and cathodic peak potentials.

To further optimal conditions of the electrochemical behavior of DA at the poly (**BTCA**)/GCE, the effect of scan rate was also investigated. The CVs (Fig. 8A) depict an enhancement in peak currents with a variation in the scan rate from 25 to 500 mV/s 35.0 μM of DA in 0.1 M PBS (pH 7.0) at the poly(**BTCA**)/GCE. Noticeably, the response of the redox peak currents correspondingly increased with the scan rate. Additionally, a plot of the peaks currents vs the square root of scan rate yields a straight lines segment as shown in Fig. 8B. The regression equations were expressed as the following equations.

$$I_{{{\text{pa}}}} \left( {\upmu {\text{A}}} \right) = 11.482\upupsilon ^{1/2} \left( {{\text{mV}}/{\text{s}}} \right)^{1/2} + 10.202 \, \left( {{\text{R}}^{2} = 0.9985} \right)$$

$$I_{{{\text{pc}}}} \left( {\upmu {\text{A}}} \right) = 8.533\upupsilon ^{1/2} \left( {{\text{mV}}/{\text{s}}} \right)^{1/2} - 6.53 \, \left( {{\text{R}}^{2} = 0.9964} \right).$$

Thereby, it suggests that the electrooxidation of DA at the poly(**BTCA**)/GCE is a diffusion-controlled electrocatalytic process as in the previous reports^68,69^.

### Influence of DA concentration

**Figure 9:**
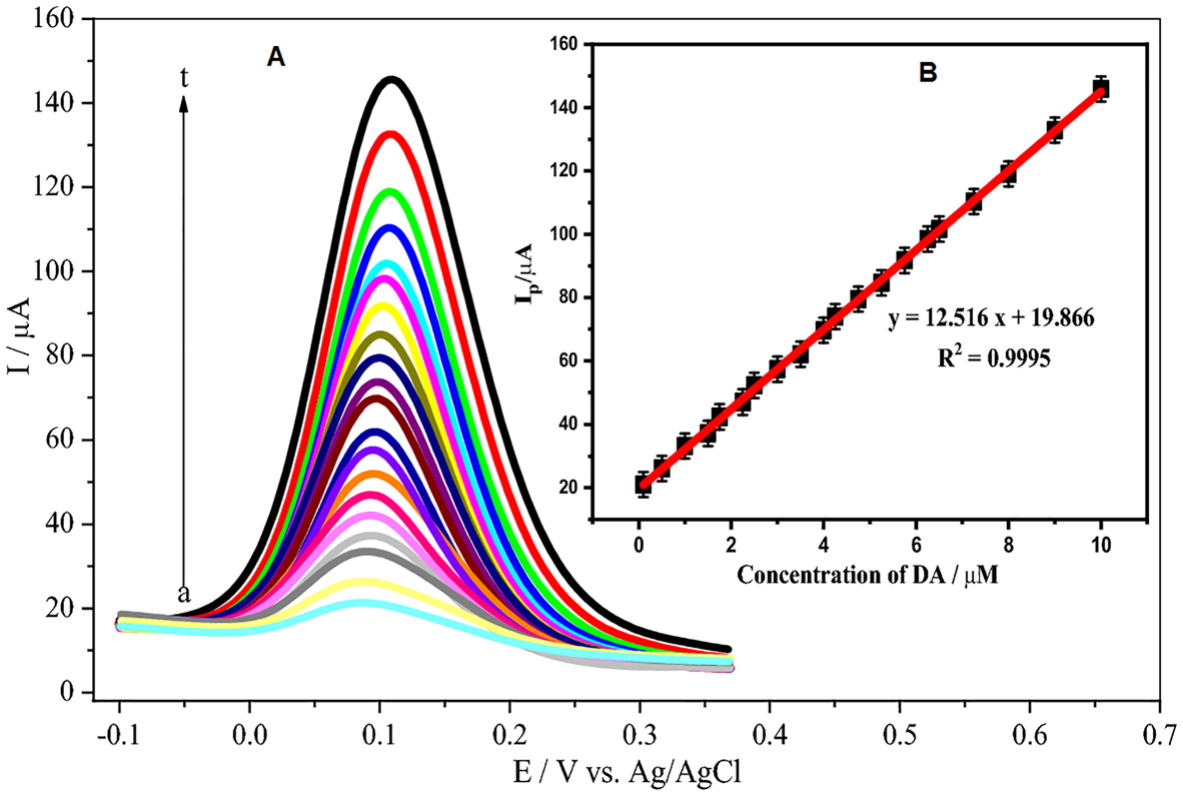
(**A**) DPVs at the poly (BTCA)/GCE for different concentrations of DA: (*a* to *t* are 0.1, 0.5, 1.0, 1.5, 1.75, 2.25, 2.5, 3.0, 3.5, 4.0, 4.25, 4.75, 5.25, 5.75, 6.25, 6.5, 7.25, 8.0, 9.0 and 10.0 µM) in 0.1 M PBS (pH 7.0). Pulse amplitude: 25 mV, pulse width: 50 ms, and scan rate: 200 mV/s. (**B**) Inset: Plot of the peak current vs. DA concentration from 0.1 to 10.0 µM.

Differential pulse voltammetry (DPV) is one of the voltammetric techniques that have high sensitivity and better resolution^70^. Thus, DPV was used to measure DA with different concentrations in the range of 0.1 to 10.0 μM in 0.1 M PBS (pH 7.0) at the poly(**BTCA**)/GCE (Fig. 9A). In this method, optimal conditions were achieved with pulse high 25 × 10^−3^ V, pulse width 50 × 10^−3^ s, step time 0.1 s and scan rate 20 mV/s. As a result, the rising DA concentration leads to an obvious increase in the anodic peak current. Therefore, inset Fig. 9B displays a calibration curve that was obtained by a plot of the anodic peak current vs DA concentration. A good linear regression equation was calibrated as

$$I_{{{\text{pa}}}} \left( {\upmu {\text{A}}} \right) = 12.516{\text{C}}\left( {\upmu {\text{M}}} \right) + 19.86\;{\text{with}}\;{\text{the}}\;{\text{correlation}}\;{\text{coefficient}}\;({\text{R}}^{2} = 0.9995)$$

The limit of detection (LOD; 3σ/m) and limit of quantification (LOQ; 10σ/m) {where (σ = Standard Deviation (S.D) of 3 replicate runs for the lowest concentration and m = the slope of calibration curve)} are 0.9995, 12.516 µA/µM, 28 nM, and 94 nM, respectively, which are superior or similar to the previously reported electrodes as shown in Table 2.

**Table 2:** Analytical parameters of several reported electrodes for the detection of DA.

Method	Modified electrode	Linear range (µM)	Sensitivity (µA/µM)	LOD (µM)	References
DPV	COF/Pt/MWCNTCOOH	2.0–500	–	0.67	^71^
DPV	CGPE^a^	0.1–2300	0.8	0.032	^72^
DPV	Ti_3_C_2_/G-MWCNTs/ZnO	0.01–30	16.0	0.37	^73^
DPV	Dual-MIP^b^	0.6–200	0.39, 6.64	0.03	^74^
DPV	Cu-BTC/CPE^c^	0.05–500	0.0937	0.03	^75^
DPV	AuNPs@TS-COF/RGO	0.5–20, 20–100	1.199	0.0628	^76^
Amp	PABA/PSS^d^	0.1–1.0	0.007	5.83	^65^
CV	CAuNE-700 nm	1.0–100	–	1.28	^77^
CV	pGO-GNP-pGO	0.1–30	5.13	0.3	^78^
SWV	MWNTs/GCE	0.5–10	5.33	0.031	^79^
DPV	ETPG^e^	0.01–5.0	–	0.029	^80^
DPV	Poly(**BTCA**)/GCE	0.1–10	12.516	0.028	This work

^a^ZnS nanoparticle decorated composite graphene paper electrode.

^b^molecularly imprinted polymer membrane.

^c^Cu-benzene-1,3,5-tricarboxylic acid.

^d^poly(3-aminobenzylamine)/poly (sodium 4-styrenesulfonate).

^e^electrochemically treated pencil graphite.

## Interference study

**Figure 10:**
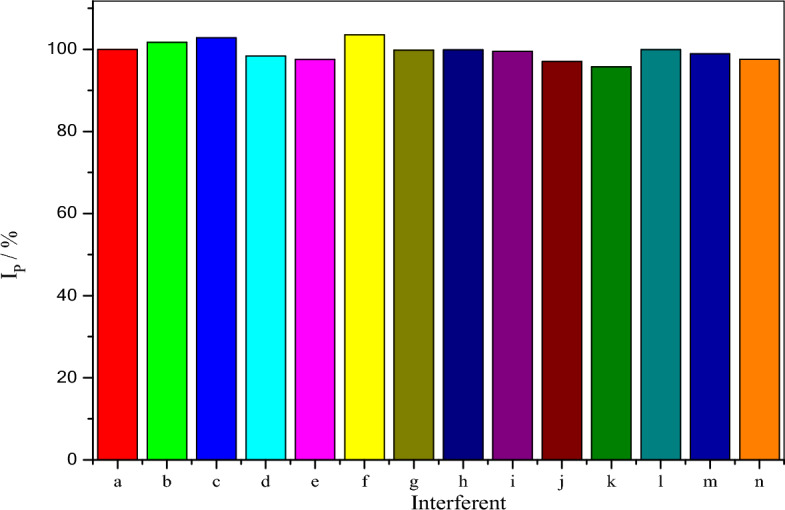
Measurements of % of *I*_pa_ of 35.0 µM DA in 0.1 M PBS (pH 7.0) at the poly(BTCA)/GCE in the absence (a) and the presence of the tolerance limit of NaCl (b) KNO_3_ (c) CaSO_4_ (d) FeCl_3_ (e) UA (f) AA (g) CA (h) TA (i) Glu (j) Lac (k) Ala (l) Cys (m) and Urea (n).

To appraise the selectivity of the poly (**BTCA**)/GCE, the influence of several possible interfering molecules on the detection of DA was investigated. Various interference species were individually added to 0.1 M PBS (pH 7.0) containing 35.0 µM of DA, and changes in the percentage of DA anodic peak current were measured as shown in Fig. 10. The results indicate that the common ions, such as Na^+^, K^+^, Ca^2+^, Fe^3+^, Cl^-^, NO_3_^-^, and SO_4_^2-^, did not show interference with DA detection. Uric acid, ascorbic acid, citric acid, tartaric acid, glucose, lactose, alanine, cysteine, and urea have no clear interference in DA determination, as summarized in Table 3.

**Table 3:** Effect of some interferences on 35.0 µM of DA.

Interferents	Tolerance limit^a^ (Mol_Ion_/Mol_RF_)
Na^+^, K^+^, Ca^2+^, Fe^3+^, Cl^-^, NO_3_^-^, SO_4_^2-^	1000^b^
Citric acid and tartaric acid	500
Glucose and lactose	500
Alanine and cysteine	100
Ascorbic acid, uric acid and urea	50

^a^The concentration gave a relative error of ± 5.0%. ^b^Maximum concentration was tested.

## Reproducibility, repeatability and stability of the poly(BTCA)/GCE

**Figure 11:**
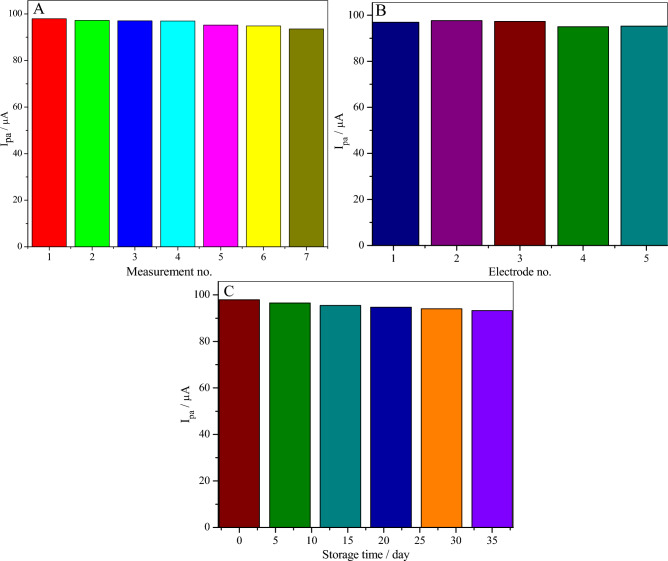
Measurements of the anodic peak current of 35.0 µM of DA in 0.1 M PBS (pH 7.0) at a scan rate of 50 mV/s for (**A**) seven times at the same poly(BTCA)/GCE, (**B**) five independent electrodes and (**C**) different storage times.

The reproducibility and repeatability of the poly (**BTCA**)/GCE towards the DA determination were investigated. The repeatability of the modified electrode was evaluated by successive measurements of seven solutions of 0.1 M PBS (pH 7.0) containing 6.0 µM of DA (Fig. S6) leading to a relative standard deviation of 1.51% (Fig. 11A). Afterwards, Fig. 11B depicts the reproducibility of the fabricated sensor by prepared five independently poly (**BTCA**)/GCE via the same conditions and used to measure 6.0 μM DA in 0.1 M PBS at pH 7.0 (RSD is 1.13%) (Fig. S7). Moreover, the stability of the fabricated sensor was also evaluated. Through four weeks, there is no significant shift observed for the oxidation peak potential of DA with a 4.73% reduction in the current (Fig. 11C) when the poly (**BTCA**)/GCE in 0.1 M PBS at room temperature (Fig. S8). Therefore, all these results indicate that the poly (**BTCA**)/GCE has good reproducibility, repeatability, and stability.

## Application in biological fluids

With negligible background current, the DPV has been applied to the detection of the DA content in real samples because of its sensitivity and selectivity^11,22^. Next, the applicability of the fabricated electrode was tested in spiked blood serum and human urine samples. Diluted biological fluids were spiked with different known concentrations of DA. Recoveries of the spiked samples were determined, as represented in Table 4. We observed that the recoveries for the DA detection at the poly (**BTCA**)/GCE are in the range of 98.0% to 102.33%, declaring that this proposed sensor is effective and reliable.

**Table 4:** Determination of DA in biological samples using the poly(BTCA)/GCE (n = 3).

Sample	DA added (μM)	Founded (μM)	Recovery (%)	RSD (%)
Blood serum	0	ND*	–	–
	1.0	0.99	99.0	4.07
	2.0	2.04	102.0	1.94
	3.0	2.98	99.3	2.81
Human urine	0	ND*	–	–
	1.0	0.98	98.0	2.17
	2.0	1.98	99.0	3.39
	3.0	3.07	102.3	1.91

*Not detected.

## Conclusion

In our work, we reported for the first time a new electrochemical sensor for the voltammetric detection of Dopamine based on the formation of a polymer film from 1,3-Benzothiazol-2-yl((4-carboxlicphenyl) hydrazono) acetonitrile on the surface of glassy carbon. The synthesized azo day structure was confirmed via different techniques. The electrochemical detection of (DA) was done using both cyclic voltammetry (CV) and differential pulse voltammetry (DPV) under the optimum conditions, such as 0.1 M PBS (pH = 7) as a supporting electrolyte with scan rate 20 mV/s and the value of Pulse amplitude and pulse width were 25 mV, 50 ms respectively. From the data obtained, the utilized modifier enhances the efficiency of the selectivity and sensitivity of the electrochemical sensor for the detection of (DA) with LOD at 28 nM, in addition to a good linearity range of 0.1 to 10.0 μM was obtained. Accordingly, poly (**BTCA**)/GCE is considered effective for the electrochemical detection of DA in biological samples with a recovery value (of 98.0–102.3%).

### Supplementary Information

Supplementary Information.

## Data Availability

All data generated or analysed during this study are included in this published article [and its supplementary information files] and available from the corresponding author on reasonable request.
