# Supplementary material for: Real samples sensitive dopamine sensor based on poly 1,3-benzothiazol-2-yl((4-carboxlicphenyl)hydrazono)acetonitrile on a glassy carbon electrode
Source: Sci Rep. 2024 Jul 18;14:16601. doi: 10.1038/s41598-024-65192-0 (PMC11258363; doi:10.1038/s41598-024-65192-0)
Supplement: Supplementary file 1 — Supplementary Information. [file 41598_2024_65192_MOESM1_ESM.docx]

**Supplementary Material**

**Real Samples Sensitive Dopamine Sensor Based on Poly 1,3-Benzothiazol-2-yl((4-carboxlicphenyl)hydrazono)acetonitrile on a Glassy Carbon Electrode**

Hesham M. Alsoghier^1*^, Mohamed Abd-Elsabour^2^, Abdulrahman G. Alhamzani^3^, Mortaga M. Abou-Krisha^3^, Hytham F. Assaf^1^

^1^Chemistry Department, Faculty of Science, South Valley University, 83523 Qena, Egypt.

*^2^Chemistry Department, Faculty of Science, Luxor University, Luxor 85951, Egypt.*

*^3^Chemistry Department, College of Science, Imam Mohammad Ibn Saud Islamic University (IMSIU), Riyadh 11623, Saudi Arabia.*

***^*^Corresponding author***

****e-mail:*** [hesham.abdelreheam@sci.svu.edu.eg](mailto:hesham.abdelreheam@sci.svu.edu.eg)  ***(*Hesham M. Alsoghier*)***

Fig. S1: The ^1^H-^1^H COSY NMR spectrum of the dye (**BTCA Dye**) in DMSO-d6.

Fig. S2: The ^13^C DEPT-135 NMR spectrum of the dye (**BTCA Dye**) in DMSO-d6.

Fig. S3: The ^1^H-^13^C HSQC NMR spectrum of the dye (**BTCA Dye**) in DMSO-d6

Fig. S4: The ^1^H-^13^C HMBC NMR spectrum of the dye (**BTCA Dye**) in DMSO-d6


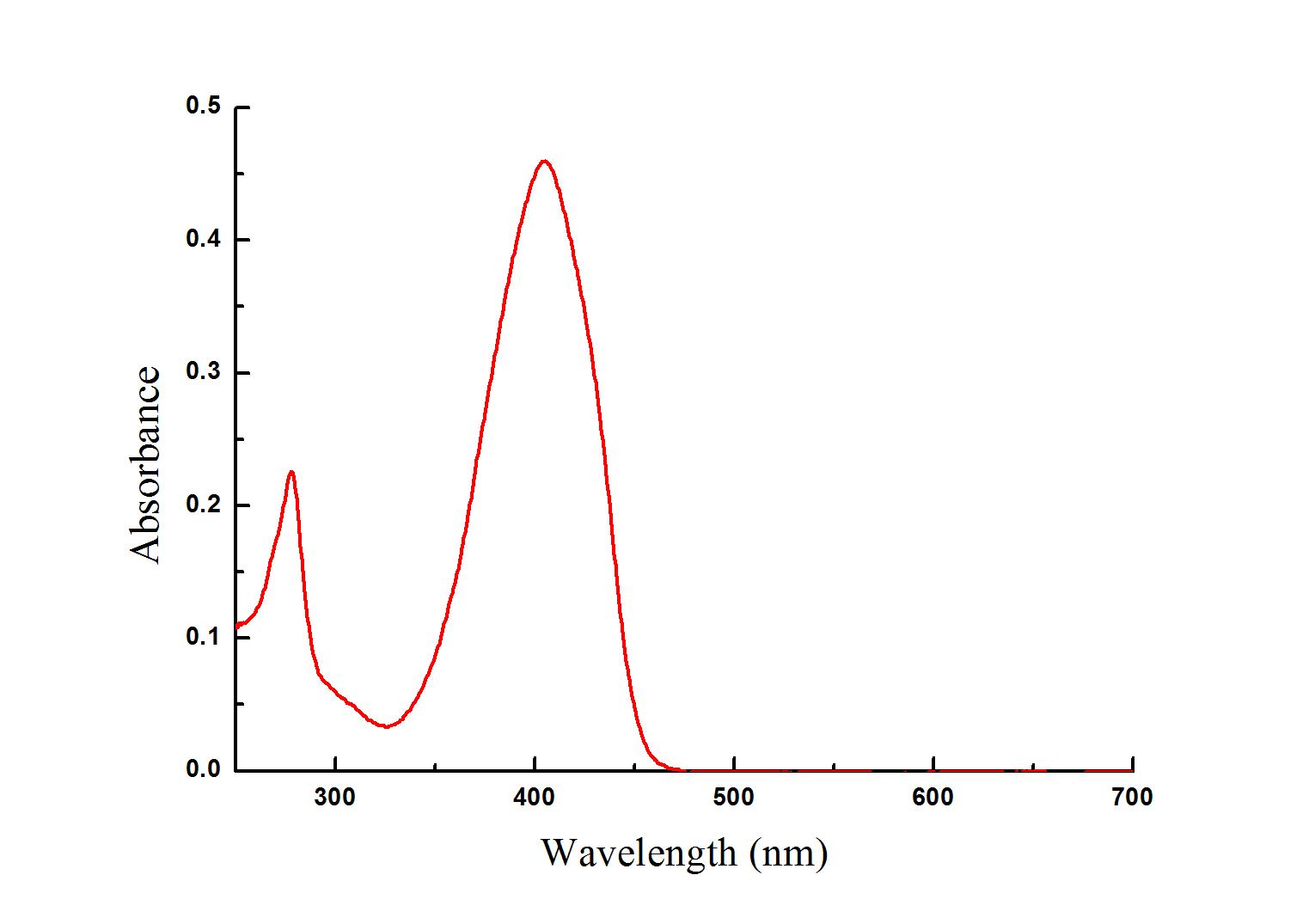


Fig. S5: The UV-Vis spectrum curve of **BTCA Dye** (2 × 10^-5^ M in CHCl_3_).

Fig S6. Six duplicates of 6.0μM DA in 0.1 M PBS at pH 7.0 in at poly(BTCA)/GCE. For the six subsequent assays, the relative standard deviation (RSD) was 1.51%.

Fig S7. DPVs of 6.0μM DA in 0.1 M PBS at pH 7.0 at the fabricated sensor by prepared five independently poly (**BTCA**)/GCE via the same conditions (RSD is 1.13%).

Fig. S8. Measurements of the anodic peak current of 6.0µM of DA in 0.1 M PBS (pH 7.0) at different storage times.


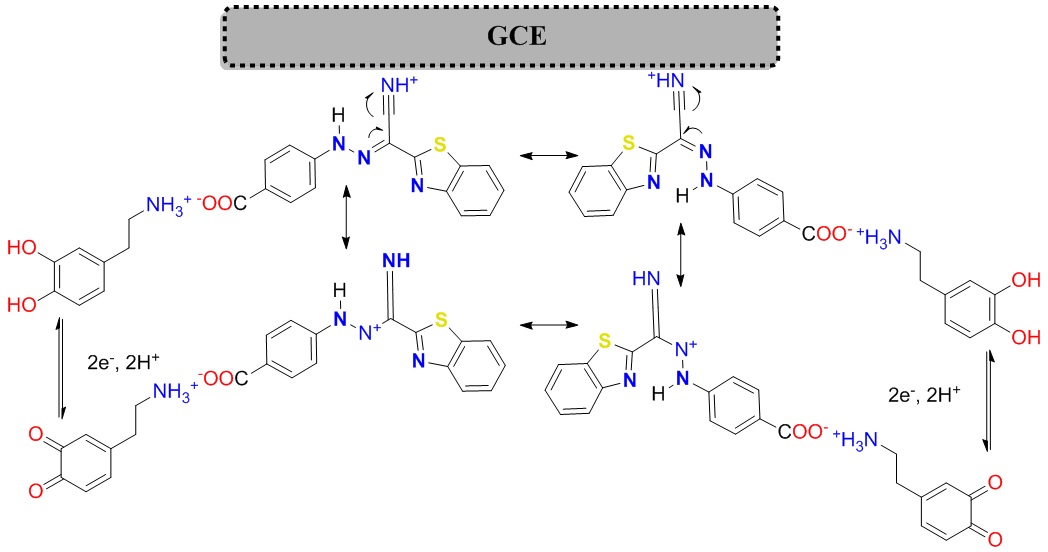


**Scheme. 3. Suggested oxidation of Dopamine (DA) on poly(BTCA)/GCE surface. (in large Scale )**
